# A longitudinal exploration of self‐perception, mental images of the self, and depression in young people

**DOI:** 10.1002/jcv2.70095

**Published:** 2026-02-10

**Authors:** Rebecca L. Dean, Victoria Pile, Ellen J. Thompson, Kathryn J. Lester, Faith Orchard

**Affiliations:** ^1^ School of Psychology, Faculty of Science, Engineering and Medicine University of Sussex Brighton UK; ^2^ Department of Psychology Institute of Psychiatry, Psychology & Neuroscience King's College London London UK; ^3^ School of Psychology University of East Anglia, Norwich Research Park Norwich UK

**Keywords:** adolescence, depression, longitudinal, mental imagery, self‐perception

## Abstract

**Background:**

Cognitive theories suggest that negative self‐perception is central to the development and maintenance of depression. One way self‐perception is represented is through mental imagery of the self. Despite its theoretical importance, the role of mental images of the self in depression has not been systematically examined. This study investigated cross‐sectional and longitudinal associations between self‐perception, mental images of the self, and depressive symptoms in young people.

**Methods:**

A total of 796 young people (aged 12–24) recruited from school and university populations completed surveys at two timepoints, 1 month apart. Measures included the Harter Self‐perception Profile Global Self‐Worth subscale, the Mental Imagery Questionnaire for Youths and the Revised Child Anxiety and Depression Scale‐Short Version.

**Results:**

Self‐perception was negatively associated with depressive symptoms both cross‐sectionally and longitudinally (b = −2.57, 95% CI [−2.87, −2.27]; b = −0.83, 95% CI [−1.22, −0.45]). Positive and negative mental images of the self were associated with depressive symptoms (*t* = −5.61; *t* = 10.73). Frequency of positive and negative images of the self were associated with self‐perception (b = −0.57, 95% CI [−0.76, −0.39]; b = 0.38 95% CI [0.18, 0.58]) and depression (b = 0.12, 95% CI [0.09, 0.16]; b = −0.10, 95% CI [−0.14, −0.06]), cross‐sectionally. Positive imagery vividness was linked to self‐perception in the full sample (b = 0.09, 95% CI [0.01, 0.16]) and school subgroup (b = 0.14, 95% CI [0.03, 0.24], but not in the university subgroup. Longitudinally, both frequency and vividness of positive imagery were associated with self‐perception in the university subgroup only (b = 0.05, 95% CI [0.01, 0.10]; b = 0.09, 95% CI [0.01, 0.16]).

**Conclusion:**

Young people's self‐perceptions, whether expressed through evaluative thoughts or mental images, play a critical role in depressive symptoms. Findings support cognitive models of depression and highlight self‐perception as a promising intervention target. The study also underscores limitations of current mental imagery measures and the need for more robust tools to clarify these relationships.

## Key points

**What's Known**

- Self‐perception plays an important role in depression; however, the nature of this relationship is unclear.
- The self is represented through mental imagery, but mental images of the self have not been explicitly studied in relation to depressive symptoms.
- Vividness and frequency of mental images have been highlighted as potentially influential in the experience of depressive symptoms.

**What's New**

- A significant longitudinal relationship was identified between self‐perception and depressive symptoms. Experiencing a mental image of the self may be a protective (positive images) or vulnerability (negative images) factor for depression.

**What's Relevant**

- Self‐perception could be an important target in interventions for depression in young people. Mental images of the self may be an important feature of depression.

## INTRODUCTION

Adolescence and early adulthood are periods of particular vulnerability for the development of mental health difficulties such as depression (Kertz et al., 2019). Three‐quarters of all lifetime cases of mental health conditions develop prior to the age of 24, with first incidence occurring most frequently at 15 years old (Kessler et al., 2005; McGrath et al., 2023). Amongst adolescents aged 10–19 years old, it is estimated that 8% meet diagnostic criteria for major depressive disorder and 34% experience elevated symptoms of depression (Shorey et al., 2022). Onset of depression during adolescence is associated with persistent challenges, including impaired social functioning, academic and employment difficulties, and higher risk of future mental and physical health problems (Clayborne et al., 2019; Johnson et al., 2018). It is therefore imperative that symptoms are recognised early, and that individuals have access to early or preventative interventions (Petito et al., 2020). However, current psychological treatments for depression have limited efficacy for young people (Eckshtain et al., 2020). One key reason for this might be that interventions are often developed for adults without specific developmental adaptations that address the concerns and challenges associated with this age group (M. Y. Ng & Weisz, 2016). Understanding the psychological mechanisms that contribute to the development and maintenance of depression in young people is therefore crucial to design more effective strategies for intervention.

Dysfunctional representations of the self are implicated in many theoretical models of depression. Beck's cognitive model proposes that depression is characterised by persistent negative thoughts about the self, driving feelings of hopelessness and worthlessness (Beck, 1967). Similarly, the self‐memory system model theorises that self‐perception influences the valence and content of autobiographical memories retrieved, and that individuals who perceive themselves negatively have cognitive biases that increase the accessibility of negative autobiographical memories over positive memories (Conway, 2005). Both mechanisms will reduce mood. Cross‐sectional research has consistently supported the link between negative self‐perception and increased low mood (Hards et al., 2023; Orchard et al., 2019). Longitudinal associations between self‐perception and depression have also been found, including evidence from a recent study which used an intensive sampling approach to measure self‐referential thinking and depression (Weisenburger et al., 2024). The researchers measured these variables in three samples of university students either daily over 5 or 7 days, or weekly over 7 weeks. The results indicated that increases in negative self‐referential thoughts were associated with increases in depressive symptoms in the 5‐day and 7‐week samples. However, this was not replicated in the 7‐day sampling group. This initial evidence suggests an important relationship between self‐perception and depression, but further research is needed to confirm this across wider age ranges so we can better understand how these variables change over time.

Current psychological treatments for depression often target negative self‐perception, either directly or indirectly. For example, cognitive behavioural therapy (CBT) may target negative self‐perception by trying to challenge negative automatic thoughts. However, this is often part of a larger treatment package that includes other focuses, and improving self‐perception may not always be a priority (Orchard et al., 2021). A recent meta‐analysis examined the change in self‐perception following interventions for depression in young people (Dean et al., 2024). Twenty studies were identified, and interventions included CBT, cognitive training, rational emotive behaviour therapy, and interpersonal therapy. Results indicated that self‐perception is sensitive to change even when not explicitly targeted. However, effect sizes for self‐perception were small, suggesting that self‐perception could be more strongly targeted with innovative treatments to improve depression in young people.

Cognitive models have traditionally focused on verbal processes to understand depression. However, mental imagery, is a key part of our inner experience that is not currently captured by measures of self‐perception (Holmes et al., 2016; Kosslyn et al., 2001; Pearson et al., 2015). Mental imagery refers to the experience of sensory representations in the mind without external stimuli, such as visualising memories or simulating future events (Holmes et al., 2016). Mental imagery can elicit emotional responses more strongly than verbal thoughts, and as such may have a greater impact on mood than verbal cognitions (Bär et al., 2023). Representations of the self in mental imagery can impact a person's self‐perception, with negative self‐representations contributing towards depression (Blackwell, 2023; D’Argembeau, 2021; Stopa & Beck, 2021). It should be noted that most measures examining mental imagery have not been robustly evaluated and therefore their reliability and validity are unclear (McIntyre et al., 2024). Furthermore, to our knowledge, there were no measures examining mental images of the self at the time of planning this study.

Individual differences in the experience of mental imagery (more generally), such as vividness and frequency, have been identified as being important in connection with depression (Holmes et al., 2016). Research has shown that depression in adolescents is associated with increased frequency and vividness of negative mental imagery and deficits in the frequency and vividness of positive mental imagery (Pile & Lau, 2018). It is possible that these aspects of mental imagery could influence the relationship between self‐perception and depressive symptoms. A systematic review incorporating consultation with young people with lived experience of anxiety and depression examined mental imagery‐based interventions and the factors influencing their effectiveness (Pile et al., 2021). Young people emphasised the use of self‐generated imagery over being asked to imagine pre‐specified imagery, as being important to treatment and warranting further examination. A systematic review by Schwarz et al. (2020) identified three studies examining mental imagery in the context of depression in adolescents, however all of these looked at cross‐sectional associations (Kuyken & Howell, 2006; Meiser‐Stedman et al., 2012; Pile & Lau, 2018). To date, no longitudinal observational studies of mental imagery in youth depression have been published, and there is very limited work investigating the self as part of mental imagery. Given that cross‐sectional research in people aged 12–60 has found that the experience of mental imagery changes across the lifespan and becomes less vivid with age (Gulyás et al., 2022), it is particularly important to investigate mental imagery within young people as it may have a stronger impact on their lives. Researchers have therefore highlighted the need for longitudinal studies to establish how the relationship between mental imagery and depression develops over time in young people (Holmes et al., 2016; Schwarz et al., 2020; Weßlau & Steil, 2014). By focusing on mental images of the self, we aim to understand their role in the development and maintenance of depression in young people, as well as their relationship with self‐perception. This knowledge could help to determine whether mental images of the self and self‐perception are promising targets for intervention in the prevention of depression in young people.

### Study aims and hypotheses

The study aims were as follows:

1. Confirm a cross‐sectional and longitudinal relationship between self‐perception and depression in young people aged 12–24 years old.
2. Determine whether there is a relationship between experiencing a positive or negative mental image of the self and depressive symptoms.
3. Understand whether frequency of experiencing a positive or negative mental image of the self is associated with symptoms of depression and self‐perception cross‐sectionally and longitudinally.
4. Test whether the vividness of an image is associated with self‐perception and depression cross‐sectionally and longitudinally in participants who do experience a mental image of themselves.
5. Understand whether either vividness or frequency of mental imagery of the self (positive or negative) has a moderating role in the relationship between self‐perception and depression.

Given that gender, ethnicity, and anxiety are known to be associated with depressive symptoms, we included these as covariates in models (Daly, 2022; Wittchen et al., 2000).

Based on previous research, we expected that self‐perception would be negatively associated with depressive symptoms at baseline (cross‐sectionally) and follow‐up (longitudinally). We predicted that there would be a significant association between experiencing a negative mental image of the self and higher levels of depressive symptomology, and the opposite for experiencing positive images of the self. We predicted that the frequency and vividness of negative mental images of the self would be positively associated with both depression and self‐perception scores, and that the opposite would be true of positive mental images of the self. In addition, we expected to identify the same associations longitudinally. Finally, we hypothesised that positive and negative imagery vividness and frequency would moderate the association between baseline self‐perception and follow‐up depressive symptoms.

## MATERIALS AND METHODS

### Participants

**FIGURE 1:**
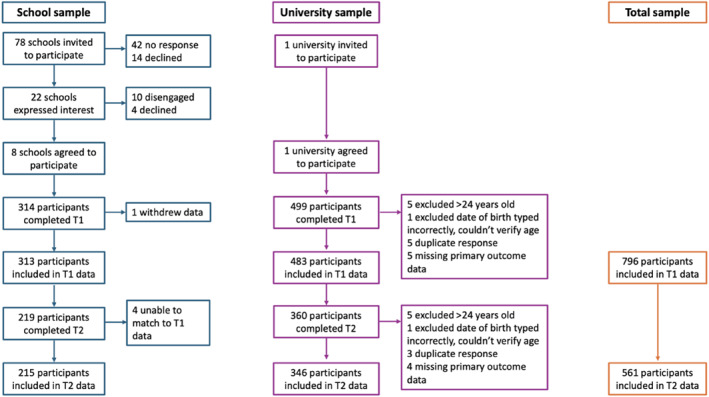
Participant flow diagram.

Data were collected at baseline from 796 young people aged 12–24 years old across two different participant groups: school students (*n* = 313, aged 12–19) and university students (*n* = 483, aged 18–24). The follow‐up survey was completed by 561 participants, with an attrition rate of 29.52% (see Figure 1).

### Measures

#### Symptoms of depression and anxiety

The Revised Child Anxiety and Depression Scale‐Short Version (RCADS‐25) is a self‐report measure of youth anxiety and depression, containing 25‐items rated on a scale from 0 (never) to 3 (always). The school sample completed the RCADS‐25 (Ebesutani et al., 2012) and university students completed the RCADS‐25 for adults, which has slightly different wording for five items to make them more applicable to adults (McKenzie et al., 2019). The RCADS‐25 has been found to have good reliability and validity in school‐based samples (Ebesutani et al., 2017). Within the present study, there was excellent internal consistency across the school sample (depression, *α* = .91 and anxiety, *α* = .90), and good internal consistency for the university sample (depression, *α* = .89 and anxiety, *α* = .86). Responses with <8 out of 10 items completed on the depression subscale and 13 out of 15 items on the anxiety subscale were considered missing. Mean replacement was used to adjust scores where fewer items were missing. *T*‐scores were calculated for the purposes of comparing our sample with population norms based on the raw score, school year, and gender; a *t*‐score of 70 or higher indicates that the respondent is experiencing symptoms above the clinical threshold for anxiety or depression, and scores of 65–69 are considered borderline clinical. Raw RCADS scores were used in all analyses.

#### Self‐perception

The school sample completed an adapted version of the Harter Self‐perception Profile for Adolescents—Global Self‐Worth subscale to measure participant's perceptions of themselves (Harter, 2012; Wichstraum, 1995). This adapted version of the scale uses a question format that is easier for participants to understand, and has improved reliability, convergent validity and factorial validity over the original version (Wichstraum, 1995). Participants were presented with five statements (e.g., ‘some teenagers are very happy being the way they are’) and were asked to rate how well it described them from ‘describes me very poorly’ to ‘describes me very well’. Items are scored from 1 to 4, and a higher score indicates increased happiness with oneself.

In the university sample the Harter Self‐Perception Profile for College Students Global Self‐Worth subscale was used to ensure the measure was appropriate for this age group, and the same question format was used (Neeman & Harter, 2012). This scale contains six items, and therefore *Z*‐scores were used in analyses to make scores comparable across both versions of the measure. Responses with less than four completed items were considered missing from the dataset for both versions. Internal consistency was good across both the university and school samples (*α* = .88 and *α* = .84 respectively).

#### Mental images of the self

The Mental Imagery Questionnaire for Youths (MIQ‐Y) was translated from German to assess characteristics of mental images (Schwarz & Schreiber, 2016) (see Appendix S1 for more information). This questionnaire was originally developed based on the Imagery Interview, which has been found to be a valid measure of imagery in adults (Hackmann et al., 2000). We altered the wording of the questionnaire to focus specifically on mental images *of the self*. Participants were first presented with a description of mental images of the self: ‘Mental images are pictures that most people can see in their “mind's eye”—like photos or movies’. These are sometimes based on real memories. But the contents of these images can also be very strange and do not necessarily have to have happened to you in that way (made‐up images). Images can refer to past events, the present, or the future. They can be pleasant, unpleasant, or even excruciating. Sometimes they show up even if you don't want them to. Many people have these mental images—some rarely, some often’. Examples of a positive or negative image were then presented, and participants were asked if they experienced a mental image like this of themselves. For those who responded ‘yes’, they were asked to rate the sensory characteristics, emotions associated, controllability, and distress caused by each mental image. Key variables used in the present analysis were frequency and vividness of mental images. Both variables were rated on a five‐point scale that indicated the extent to which participants experienced the mental image, with vividness rated from ‘no mental image at all’ to ‘perfectly clear and as vivid as in real‐life’, and frequency from ‘never’ to ‘always’. Participants who responded ‘no’ to experiencing either a positive or negative mental image of themselves skipped these questions, and responses were imputed as ‘never’ for frequency to ensure that the experiences of participants who did not have these mental images were represented in the data. As per our pre‐registered analysis plan, we had initially intended to also impute ‘no mental image at all’ for vividness as this is typically how vividness questionnaires are conceptualised in the literature (e.g., Marks, 1973), however we chose not to do this due to the large number of participants who reported not experiencing a mental image of themselves and concerns whether it was meaningful to analyse the vividness of images that have never been experienced. For analyses including vividness as an independent variable, we therefore only examined participants who reported experiencing a mental image of themselves.

#### Use of mental imagery in daily life

The Spontaneous Use of Imagery Scale (SUIS) measures participant's use of mental imagery in their daily life (Kosslyn et al., 1998). In this study participants completed a condensed 7‐item version of the SUIS adapted for adolescents (Voogd et al., 2017). Participants are presented with situations in which they might use imagery and are asked to rate how often a mental image would come to mind in the given scenario on a scale from 1 (never) to 5 (always). A higher score indicates more frequent use of mental imagery in daily life. Internal consistency was acceptable within the school sample (*α* = .71) and questionable for the university sample (*α* = .64) (see Table S1).

### Procedures

The study was approved by the University of Sussex Science and Technology Cross‐Schools research ethics committee (ER/RD416/4). There were two age‐dependent consent procedures: participants aged 16 or older were able to consent to participation themselves, whereas those under 16 years old required written consent from a responsible adult before providing their own assent. Surveys including all measures were administered at two timepoints (baseline and 1‐month follow‐up) via the Qualtrics online survey platform. Participants were able to skip any questions that they did not want to answer.

#### School sample

Participants in the school sample were students in years 8–13 attending mainstream secondary schools and sixth form colleges in England. Opportunity sampling was used to recruit eight institutions, who facilitated the study by offering it to their students either in classrooms or online at home, depending on their preference. Schools distributed the study information and consent forms to parents/carers and students via bulletins or mailing lists in advance of survey administration. A prize draw for one of five £30 vouchers was offered as an incentive for completing the second survey.

#### University sample

The university sample was comprised of students at the University of Sussex, England, recruited via the Sona participant recruitment platform. Participants were given research credits as an incentive for completion of each survey. Given that our study aimed to understand depressive symptoms in young people, we excluded data from participants aged 25 and older from the analysis in accordance with recent global definitions of youth (World Health Organization, 2024).

### Statistical analyses

The analyses were pre‐registered on the Open Science Framework (osf.io/yxh54/). To test our hypotheses, general linear modelling was utilised to look at associations between self‐perception, mental images of the self, and depression using maximum likelihood estimation. We included a random effect of institution within models to account for the non‐independence of students within academic institution using the ‘lme4’ and ‘lmerTest’ packages in RStudio (Bates et al., 2024; Kuznetsova et al., 2020). Missing data were handled based on each measure's guidance, unless otherwise specified in the measures section.

To determine whether it would be appropriate to test our hypotheses with a random effect of institution, we first specified a null model for the key outcome of depressive symptoms to determine the amount of variance caused by between‐school differences. Linear regressions were then conducted to test each hypothesis. Firstly, for each model, unadjusted associations between hypothesis‐specific predictors and outcome variables were tested. Secondly, individual level covariates were added to the model (gender, ethnicity, baseline anxiety). Given that most schools offered the study to specific year groups (see Table S2), there is limited age variability within institutions. Due to this age was not included as a covariate in models where a random fixed effect of institution was included. Some models failed to converge when the random effect of institution was added, likely due to limited power and complexity of the models. As such, some models were run without the random effect of institution, as indicated in the results tables. Where this occurred, age was added as a covariate as this was no longer precluded. Lastly, for hypotheses testing longitudinal associations, time and baseline measurements of the outcome variable being tested were added to the model. *p*‐values were adjusted using the Benjamini‐Hochberg procedure to account for multiple testing. All hypotheses were tested using the full sample, and within the university and school subgroups separately to test whether these relationships were still present across each population.

Moderation analyses were conducted to test whether characteristics of mental imagery moderated the association between baseline self‐perception and follow‐up depressive symptoms. Positive and negative mental imagery frequency and vividness scores were divided into ‘high’ and ‘low’ categories based on participant scores to maximise the power available for analysis, and input into the model as categorical predictors for the purpose of moderation analysis only. A score of three and above corresponded to a ‘high’ level of vividness or frequency, and a score of two or lower was considered ‘low’, consistent with absent or vague and dim mental imagery seen in aphantasia (Dance et al., 2022).

## RESULTS

### Demographic and clinical characteristics

Demographic characteristics and RCADS scores collected from the baseline and follow‐up surveys for both populations are presented in Tables 1, 2, 3. Our sample was predominantly white (83.0%) and female (73.9%). Over a quarter of participants (28.5%) had clinically significant depressive symptoms at baseline, and 21% had clinically significant symptoms of anxiety based on their RCADS‐25 *t*‐scores. University students had significantly higher depression raw scores than secondary school students, *d* = −0.23, *t*(599.79) = −3.03, *p* < .01. There were no significant differences in self‐perception *Z*‐scores between the two groups, *d* = 0.01, *t*(651.04) = 0.10, *p* = .92 (see Table S3), and no significant difference in raw depression scores from baseline and follow‐up*, d* = 0.02, *t*(79.68) = 0.30, *p* = .76. The mean interval between baseline and follow‐up survey completion was 34.8 days (SD 8.55; range 26.9–60.0).

**TABLE 1:** Sample demographic characteristics.

Demographic characteristic	School sample (*n* = 313)	University sample (*n* = 483)	Whole sample (*n* = 796)
Age			
Mean (SD)	15.8 (1.52)	19.2 (1.21)	17.9 (2.13)
Median [min, max]	16 [12, 19]	19 [17, 24]	18 [12, 24]
Gender			
Female	183 (58.5%)	405 (83.9%)	588 (73.9%)
Male	122 (39.0%)	48 (9.9%)	170 (21.4%)
Non‐binary or other	5 (1.6%)	29 (6.0%)	34 (4.3%)
No response	3 (1.0%)	1 (0.2%)	4 (0.5%)
Ethnicity			
White	268 (85.6%)	393 (81.4%)	661 (83.0%)
Asian	24 (7.7%)	40 (8.3%)	64 (8.0%)
Mixed or multiple	15 (4.8%)	24 (5.0%)	39 (4.9%)
Black or other	5 (1.6%)	26 (5.4%)	31 (3.9%)
No response	1 (0.3%)	0 (0%)	1 (0.1%)

**TABLE 2:** Baseline RCADS scores.

Clinical characteristics	School sample (baseline *n* = 313, follow‐up *n* = 215)	University sample (baseline *n* = 483, follow‐up *n* = 346)	Whole sample (baseline *n* = 796, follow‐up *n* = 561)
Baseline total anxiety and depression *t*‐score			
Mean (SD)	59.3 (15.5)	62.5 (15.4)	61.3 (15.5)
Median [min, max]	56.6 [31.4, 108]	62.0 [26.6, 108]	59.3 [26.6, 108]
Missing	13 (4.2%)	10 (2.1%)	23 (2.9%)
Baseline total anxiety and depression severity^a^			
Normal	198 (63.3%)	258 (53.4%)	456 (57.3%)
Borderline	20 (6.4%)	65 (13.5%)	85 (10.7%)
Clinical	82 (26.2%)	150 (31.1%)	232 (29.1%)
Missing	13 (4.2%)	10 (2.1%)	23 (2.9%)
Baseline depression subscale *t*‐score			
Mean (SD)	57.5 (16.2)	60.3 (15.1)	59.2 (15.6)
Median [min, max]	55.9 [29.3, 131]	58.4 [29.3, 120]	57.0 [29.3, 131]
Missing	13 (4.2%)	10 (2.1%)	23 (2.9%)
Baseline depression severity^a^			
Normal	206 (65.8%)	287 (59.4%)	493 (61.9%)
Borderline	22 (7.0%)	31 (6.4%)	53 (6.7%)
Clinical	72 (23.0%)	155 (32.1%)	277 (28.5%)
Missing	13 (4.2%)	10 (2.1%)	23 (2.9%)
Baseline anxiety subscale *t*‐score			
Mean (SD)	59.5 (17.1)	63.1 (16.3)	61.7 (16.7)
Median [min, max]	55.6 [28.5, 129]	61.6 [28.5, 121]	60.2 [28.5, 129]
Missing	13 (4.2%)	10 (2.1%)	23 (2.9%)
Baseline anxiety severity^a^			
Normal	212 (67.7%)	308 (63.8%)	520 (65.3%)
Borderline	27 (8.6%)	59 (12.2%)	86 (10.8%)
Clinical	61 (19.5%)	106 (21.9%)	167 (21.0%)
Missing	13 (4.2%)	10 (2.1%)	23 (2.9%)

^a^ Severity classification was based on participant's *t*‐scores using the following thresholds: clinical ≥70, subclinical: 65–69, not clinically significant <65.

**TABLE 3:** Follow‐up RCADS scores.

Clinical characteristics	School sample (*n* = 215)	University sample (*n* = 346)	Whole sample (*n* = 561)
Follow‐up total anxiety and depression *t*‐score			
Mean (SD)	58.3 (14.5)	62.2 (15.0)	60.8 (14.9)
Median [min, max]	56.0 [26.6, 97.4]	62.0 [29.3, 108]	59.3 [26.6, 108]
Missing	8 (3.7%)	0 (0.0%)	8 (1.4%)
Follow‐up total anxiety and depression severity^a^			
Normal	142 (66.0%)	200 (57.8%)	342 (61.0%)
Borderline	19 (8.8%)	41 (11.8%)	60 (10.7%)
Clinical	46 (21.4%)	105 (30.3%)	151 (26.9%)
Missing	8 (3.7%)	0 (0.0%)	8 (1.4%)
Follow‐up depression subscale *t*‐score			
Mean (SD)	56.0 (15.3)	59.6 (14.3)	58.3 (14.7)
Median [min, max]	54.7 [26.9, 104]	58.4 [30.3, 103]	56.6 [26.9, 104]
Missing	8 (3.7%)	0 (0.0%)	8 (1.4%)
Follow‐up depression severity			
Normal	146 (67.9%)	212 (61.2%)	358 (63.8%)
Borderline	17 (7.9%)	21 (6.1%)	38 (6.8%)
Clinical	44 (20.5%)	113 (32.7%)	157 (28.0%)
Missing	8 (3.7%)	0 (0.0%)	8 (1.4%)
Follow‐up anxiety subscale *t*‐score			
Mean (SD)	58.0 (15.9)	62.4 (15.3)	60.7 (15.7)
Mean [min, max]	56.4 [24.2, 102]	61.4 [29.3, 114]	59.1 [24.2, 114]
Missing	8 (3.7%)	0 (0.0%)	8 (1.4%)
Follow‐up anxiety severity^a^			
Normal	152 (70.7%)	239 (69.1%)	391 (69.7%)
Borderline	14 (6.5%)	38 (11.0%)	52 (9.3%)
Clinical	41 (19.1%)	69 (19.9%)	110 (19.6%)
Missing	8 (3.7%)	0 (0.0%)	8 (1.4%)

^a^ Severity classification was based on participant's *t*‐scores using the following thresholds: clinical ≥70, subclinical: 65–69, not clinically significant <65.

### Characteristics of mental imagery

Over two thirds (baseline *n* = 535, 67.2%; follow‐up *n* = 384, 68.4%) of the sample endorsed experiencing a positive mental image of themselves and over half (baseline *n* = 492, 61.8%; follow‐up *n* = 310, 55.3%) reported experiencing a negative mental image of themselves (Table S4). Within the baseline sample, 360 participants indicated that they experienced both a positive and a negative mental image of themselves and 122 participants had neither valence of image. Of the remaining participants, 124 experienced a negative mental image of themselves but not a positive image, and 166 participants experienced only a positive mental image of themselves. At baseline, participant's chosen negative images were experienced significantly more frequently, *t*(586.79) = −2.80, *p* < .01, and significantly more vividly by university students compared to secondary school students, *t*(589.69) = −2.90, *p* < .01. There was no significant difference in vividness or frequency of the reported positive mental image between the two groups.

### Hypothesis testing

**FIGURE 2:**
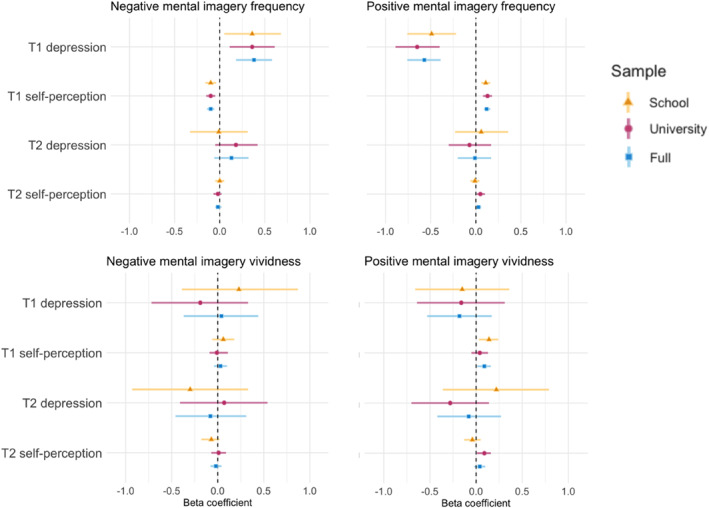
Plots representing beta values and 95% confidence intervals for each mental imagery model predicting cross‐sectional and longitudinal depression and self‐perception outcomes. T1 = baseline, T2 = follow‐up after 1 month.

The results of the main analyses are displayed in Table 4 and results from the school and university subgroup analyses are in Tables S5 and S6 respectively. Please see Figure 2 for plots representing the key mental imagery findings. Results from a likelihood‐ratio test comparing the null multi‐level model with a null single‐level model demonstrated that the multi‐level model was a better fit for the data (*χ* ^2^(1) = 9.85, *p* < .01). The intraclass correlation coefficient identified that 3.0% of variance in depression scores was explained by between‐school differences.

**TABLE 4:** Summary of models predicting depression and self‐perception.

Dependent variables		Summary of models predicting depression and self‐perception				
		Independent variables				
		Self‐perception	Negative mental image frequency	Negative mental image vividness	Positive mental image frequency	Positive mental image vividness
		b (95% CI)	b (95% CI)	b (95% CI)	b (95% CI)	b (95% CI)
Baseline	Depression	**−2.57 (−2.87, −2.27)**	**0.38 (0.18, 0.58)**	0.04 (−0.37, 0.44)*	**−0.57 (−0.76, −0.39)**	−0.18 (−0.53, 0.17)
	Self‐perception		**−0.10 (−0.14, −0.06)**	0.03 (−0.04, 0.10)	**0.12 (0.09, 0.16)**	**0.09 (0.01, 0.16)**
Follow‐up	Depression	**−0.83 (−1.22, −0.45)**	0.13 (−0.06, 0.32)	−0.08 (−0.46, 0.31)	−0.01 (−0.20, 0.17)	−0.08 (−0.42, 0.27)*
	Self‐perception		−0.02 (−0.05, 0.02)*	−0.02 (−0.08, 0.04)	0.03 (−0.00, 0.06)*	0.04 (−0.02, 0.10)*
Moderation follow‐up	Depression (self‐perception × mental image characteristic)		0.09 (−0.51, 0.69)	0.55 (−0.40, 1.50)	−0.12 (−0.69, 0.45)	−0.07 (−1.01, 0.87)*

*Note*: Models with mental imagery vividness as an independent variable use data only from participants who reported experiencing a mental image of themselves and models with mental image frequency use imputed data. Baseline models were adjusted using gender, ethnicity, and anxiety. Follow‐up models were further adjusted using time and baseline outcome. Models include a random effect of school unless indicated with *, and these models are also adjusted by age. Coefficients in bold are where the 95% CIs do not cross the null. Beta coefficients are unstandardised, except where self‐perception was included in the models due to use of *Z*‐scores.

#### Cross‐sectional and longitudinal relationships between self‐perception and depression

Linear regression was employed to examine whether self‐perception was associated with depressive symptoms. At baseline, self‐perception scores were negatively associated with depression scores (b = −2.57, *t*(768.95) = −16.85, *p* < .01), indicating that poorer self‐perception is linked to higher levels of depressive symptoms. Longitudinally, baseline self‐perception scores were found to significantly negatively predict depressive symptoms at follow‐up when controlling for baseline depression (b = −0.83, *t*(549.54) = −4.25, *p* < .01), suggesting that individuals with poorer self‐perception experienced higher levels of depressive symptoms 1 month later.

When these relationships were investigated in each group separately, the cross‐sectional associations remained significant in the school (b = −2.20, *t*(294) = −8.65, *p* < .01) and university samples (b = −2.76, *t*(463) = −14.52, *p* < .01). However, the longitudinal relationships were only significant in the university sample (b = −0.96, *t*(336) = −4.01, *p* < .01).

#### Relationships between experiencing a positive or negative mental image of the self and depressive symptoms

Independent samples *t*‐tests were run to test the association between experiencing a specific valence of mental image of the self and depressive symptoms. Results showed that young people who experienced a negative mental image of themselves experienced more depressive symptoms (*M* = 14.29, SD = 5.98) than those who did not (*M* = 9.83, SD = 5.33) (*t*(652.99) = 10.73, *p* < .01) in the baseline sample. These findings were also replicated at the follow‐up timepoint (yes: *M* = 14.61, SD = 5.73; no: *M* = 9.84, SD = 4.81), (*t*(550.55) = 10.65, *p* < .01).

Conversely, individuals who experienced a positive mental image of themselves had lower levels of depression (*M* = 11.76, SD = 5.64) than those who did not experience a positive mental image of themselves within the baseline sample (*M* = 14.55, SD = 6.75) (*t*(411.77) = −5.61, *p* < .01). Similar findings were present in the follow‐up sample (yes: *M* = 11.90, SD = 5.59; no: *M* = 13.69, SD = 6.14) (*t*(298.64) = −3.26, *p* < .01). These findings indicate that experiencing a mental image of the self may be a vulnerability (negative images) or protective (positive images) factor for depression.

When the relationship between negative mental imagery and depression was examined by subgroup, significant associations were found between experiencing a negative mental image and depressive symptoms within the baseline sample in school (yes: *M* = 14.31, SD = 6.44; no: *M* = 8.64, SD = 5.04) (*t*(295.92) = 8.52, *p* < .01) and university students (yes: *M* = 14.28, SD = 5.73; no: *M* = 10.84, SD = 5.39) (*t*(320.18) = 6.38, *p* < .01). Within the follow‐up sample, a significant association was between experiencing a negative mental image and depressive symptoms for both the school population (yes: *M* = 14.44, SD = 5.95; no: *M* = 9.07, SD = 4.98) (*t*(185.97) = 7.01, *p* < .01) and university population (yes: *M* = 14.69, SD = 5.64; no: *M* = 10.51, SD = 4.57) (*t*(318.85) = 7.53, *p* < .01).

Similarly, the relationship between positive mental imagery and depression was tested within each subgroup. Significant associations between experiencing a positive mental image of the self and reduced depressive symptoms were identified in the baseline sample for the school subgroup (yes: *M* = 10.79, SD = 5.75; no: *M* = 13.65, SD = 7.42) (*t*(181.33) = −3.46, *p* < .01) and university subgroup (yes: *M* = 12.31, SD = 5.52; no: *M* = 15.25, SD = 6.11) (*t*(233.54) = −4.89, *p* < .01). A significant association was also found at the follow‐up timepoint for the university sample (yes: *M* = 12.27, SD = 5.49; no: *M* = 14.91, SD = 5.55) (*t*(185.26) = −4.04, *p* < .01). However, no significant association was found at follow‐up in the school sample (*t*(120.60) = −0.71, *p* = .48).

#### Cross‐sectional and longitudinal associations between frequency of mental images of the self and self‐perception and depressive symptoms

In order to test whether how often young people experienced a positive or negative mental image of the self was associated with symptoms of depression or self‐perception, linear regression analyses were employed using the full sample data (*n* = 796). This included imputed responses from participants who reported they did not experience a particular mental image of themselves. We counted these responses as ‘never’ for frequency to ensure that the experiences of participants who did not experience a mental image were represented in the data.

Cross‐sectional analyses identified that frequency of experiencing a negative mental image of the self was positively associated with depressive symptoms (b = 0.38, *t*(755.93) = 3.83, *p* < .001), whilst frequency of experiencing a positive mental image was significantly negatively associated with depressive symptoms (b = −0.57, *t*(759.59) = −6.19, *p* < .001). These findings held within both populations separately for both negative mental image frequency (school subgroup: b = 0.36, *t*(282.60) = 2.28, *p* = .02; university subgroup: b = 0.36, *t*(465) = 2.78, *p* < .01) and positive mental image frequency (school subgroup: b = −0.49, *t*(284) = −3.55, *p* < .01; university subgroup: b = −0.65, *t*(464) = −5.22, *p* < .01).

Cross‐sectionally, frequency of a participant's negative mental image of the self was significantly negatively associated with self‐perception (b = −0.10, *t*(753.29) = −4.98, *p* < .001), and frequency of positive mental image of themselves was positively associated with self‐perception (b = 0.12, *t*(755.38) = 6.57, *p* < .001). Significant associations were found in each subgroup for negative mental image frequency (school subgroup: b = −0.10, *t*(282.40) = −3.16, *p* < .01; university subgroup: b = −0.10, *t*(462) = −3.85, *p* < .01) and positive mental image frequency (school subgroup: b = 0.11, *t*(286.95) = 4.04, *p* < .01; university subgroup: b = 0.13, *t*(462) = 5.20, *p* < .01).

Longitudinal analyses found that baseline frequency of both positive and negative mental images of the self did not significantly predict either depressive symptoms or self‐perception at follow‐up in the combined sample. However, within the university sample, baseline positive mental imagery of the self was a significant predictor of self‐perception at follow‐up (b = 0.05, *t*(332) = 2.57, *p* = .01). This relationship was not significant in the school sample, and no other significant longitudinal relationships between mental image frequency and self‐perception or depression were identified in the subgroup analyses.

#### Associations between vividness of mental images of the self and self‐perception and depressive symptoms

Linear regression was conducted to assess the relationships between the vividness of mental imagery and depression and self‐perceptions within participants who experienced a mental image of themself.

No significant cross‐sectional relationships were found between the vividness of participant's positive or negative mental image of the self and depression in the combined sample or either subgroup. However, there was a significant relationship between the vividness of a positive mental image of the self and baseline self‐perception in the combined sample (b = 0.09, *t*(499.28) = 2.38, *p* = .02) and school subgroup (b = 0.14, *t*(174.18) = 2.62, *p* < .01), but not within the university sample.

Analyses did not identify any significant longitudinal relationships between vividness of either a positive or negative mental image of the self and depression or self‐perception in the combined sample or school subgroup. In the university subgroup, a significant longitudinal relationship was found between the vividness of a participant's positive mental image of themselves and self‐perception 1 month later (b = 0.09, *t*(237) = 2.14, *p* = .03).

#### Vividness and frequency of mental images as moderators between self‐perception and depression

Moderation analyses were conducted to explore whether the vividness or frequency of participant's positive or negative mental imagery moderated the relationship between baseline self‐perception and follow‐up depressive symptoms. No moderation models were significant (Table 4).

## DISCUSSION

This study aimed to investigate the cross‐sectional and longitudinal relationships between depressive symptoms, self‐perception and frequency and vividness of mental imagery in young people. Our results demonstrate that self‐perception was significantly negatively associated with baseline depression scores, supporting the well‐established link between negative self‐perception and depressive symptoms (Beck, 1967; Hards et al., 2023; Orchard et al., 2019). This study builds upon previous evidence by establishing a longitudinal relationship between self‐perception and symptoms of depression. This was found within the main analysis and university subgroup but not within the school subgroup. Overall, young people with negative views of themselves were significantly more likely to experience depressive symptoms at 1 month follow‐up, evidencing both a concurrent and longitudinal effect of negative self‐perception on depression. This evidence is consistent with current understandings of the role of self‐perception in the development of depression, highlighting that changes in self‐perception are linked to future changes in depression over the course of 1 month (Weisenburger et al., 2024). Given changes were identified over this relatively short follow‐up period, this suggests that self‐perception may have a fast‐acting and powerful impact on mood in young people, particularly within young adults.

Our results demonstrate that experiencing a positive mental image of the self was associated with lower levels of depression, and experiencing a negative mental image of the self was associated with higher levels of depression. These results suggest that the experience of mental images of the self is linked with mood, and the valence of these mental images may influence vulnerability either towards (if negative images are present) or protection against (if positive images are present) depression. These findings add to the literature by demonstrating that view of self can be expressed in many modalities and that this can impact depression symptoms whether through thoughts or images.

Results of this study identified cross‐sectional relationships between frequency of mental images of the self and self‐perception and depressive symptoms. At baseline, the frequency of negative mental images of the self was associated with greater depressive symptoms and lower self‐perception, whilst the inverse was true of positive mental images of the self. Previous research into social anxiety in adult populations has indicated that mental images of the self can affect self‐evaluation, and our findings expand this understanding to young people and depressive symptoms (A. S. Ng et al., 2014). The frequency findings are consistent with previous research on mental imagery more generally (e.g., imagery of events rather than of the self), which suggests that higher frequency of negative mental imagery exacerbates low mood, whilst positive mental imagery can serve a protective role against it (Holmes et al., 2016; Pile & Lau, 2018). Interestingly, a larger effect size was found for frequency of positive mental imagery and depressive symptoms than for negative mental images. Recently, there has been an increasing recognition of the role that positive affect plays in depression, including diminished positive emotion and anhedonia (Craske et al., 2024). The current study highlights the importance of considering both positive and negative mental imagery of the self in the experience of depression.

Despite the strong cross‐sectional associations observed at baseline, there was not a significant longitudinal relationship between frequency of mental images of the self (positive or negative) and symptoms of depression or self‐perception in the full sample. This may suggest that images of the self may be an important concurrent feature of depression, but do not contribute towards changes in depressive symptoms over time. Alternatively, this lack of longitudinal association may be due to the way that imagery was measured. The adapted MIQ‐Y assesses the characteristics of one positive and one negative image rather than capturing multiple mental images experienced by participants (Schwarz & Schreiber, 2016) or different indices of mental imagery. However, a significant longitudinal relationship was found between positive imagery frequency and self‐perception within the university subgroup. This implies that the role of mental images of the self may change over time, and that positive imagery may have a stronger influence on self‐perception during young adulthood, which may in turn protect against depressive symptoms. Moderation analyses were also not significant, suggesting that frequency of mental imagery of the self may not play a buffering or exacerbating role in the relationship between self‐perception and depressive symptoms over time.

The relationship between vividness within depression and self‐perception within participants who did experience a mental image of themselves was less clear. Significant cross‐sectional relationships between self‐perception and the vividness of a participant's positive mental image of the self were identified in the full sample and school subgroup, but not in the university subgroup. This may indicate that the vividness of positive mental images of the self may particularly affect self‐perception in younger adolescents but less so in young adults. Furthermore, whilst a cross‐sectional relationship was not identified in the university subgroup, a longitudinal relationship was found between baseline positive mental imagery vividness and follow‐up self‐perception in this population, demonstrating that experiencing a positive mental imagery vividly was linked with future positive self‐perception. Vividness of either valence of mental imagery was not correlated with depressive symptoms across all samples, cross‐sectionally and longitudinally. This contrasts with recent research that has found that the vividness and ease of generating positive prospective mental imagery is associated with lower depressive symptoms in young adults (Marciniak et al., 2023). The lack of significant relationship between vividness of mental imagery and depression in the present study is at odds with these findings, although our results may be influenced by not including participants who did not experience mental imagery within these analyses. However, these findings are consistent with studies within the memory literature which demonstrate differences in the vividness of autobiographical memories in formerly depressed individuals based on their current mood state compared with never depressed individuals (Werner‐Seidler & Moulds, 2011, 2012). It may be that the experience of a positive or negative mental image of the self is more important than the vividness of the image, or that imagery characteristics other than vividness (such as how a person appraises the mental image) are more important in influencing mood.

### Strengths and limitations

First, it should be acknowledged that research into mental images of the self is in its infancy and that this research is very novel and exploratory in nature. This study is therefore an early contribution to a small body of literature. The key strengths of this research were the longitudinal design and relatively large sample of almost 800 participants. Notably, over a quarter of participants (28.5%) experienced clinically significant levels of depression based on their RCADS‐25 scores, which is in line with recent global estimates of 34% (Shorey et al., 2022). Whilst cross‐sectional studies provide valuable insight into the relationship between self‐perception, mental imagery and depression at a single time‐point, they are limited in their ability to capture the evolving nature of these cognitive processes. By studying these processes longitudinally, this study offers a more nuanced understanding of how self‐perception, mental images of the self and depression relate to one another, which may support our understanding of factors driving the development of depression. In addition, this research focused specifically on mental images of the self rather than all mental imagery. Given the lack of robust mental imagery measures available (McIntyre et al., 2024), we used a novel unvalidated measure, an adapted version of the MIQ‐Y (Schwarz & Schreiber, 2016), which has the benefit of assessing self‐generated mental imagery in young people. Self‐generated mental imagery has been suggested to be important within mental‐imagery based interventions (Pile et al., 2021), and is more reflective of young people's every day mental realities than assessing their ability to generate pre‐specified mental images. A further strength was the use of the RCADS to assess anxiety as well as depression, so that this could be included in our analyses. Self‐perception and mental imagery have previously been examined in the context of anxiety, and accounting for anxiety in analyses is particularly important given the high rates of co‐morbidity in young people (Fennell, 1998; Pile & Lau, 2020; Schreiber & Steil, 2013).

Whilst this study provides some important insights, several limitations should be acknowledged. First, the follow‐up period was relatively short (1 month), which may have limited our ability to identify changes in mental images of the self and depression that occur more gradually over time. Second, our sample may lack generalisability due to sampling methods. There was a lack of variation in the ages of participants in each school, and especially a lack of younger participants aged 12–14. A large proportion of participants were female (73.9%), and this gender discrepancy was greater in the university sample which was largely drawn from psychology undergraduate students (83.9% compared with 58.5% of secondary school students). These disparities may have implications for the applicability of our results to males and younger adolescents. Third, a high proportion of young people reported that they did not experience a mental image of themselves (30.9% for positive images and 36.2% for negative images). Whilst it is possible that this is due to genuine variability in the experience of mental imagery of the self, this rate is much higher than we would expect given that aphantasia is reported to affect 3.9% of the population (Dance et al., 2022). It is unclear whether this is due to mental images of the self being difficult for young people to retrieve, or whether it could be due to the measure and prompts used. Online studies using the MIQ‐Y have found a high non‐completion rate (Schwarz et al., 2021; Schwarz & Stangier, 2023). The authors of these studies used forced‐choice responses to eliminate missing data, whilst in our survey we allowed participants to skip items, which could have led to more people who didn't experience mental imagery completing the study rather than dropping out. However, since participants who indicated that they did not experience mental imagery were able to skip all items relating to characteristics of mental images, this may have created a response bias as it reduced the time spent on the survey. Furthermore, whilst the use of a novel measure was necessitated by the lack of validated mental imagery measures available (McIntyre et al., 2024), this created challenges in understanding how to treat the data and comparability with other measures of mental imagery. Whilst it is technically possible for respondents to score as having ‘no mental image’ on other measures such as the Prospective Imagery Task (MacLeod & Byrne, 1996), in practice this is very unlikely because vividness is assessed using multiple items. In contrast, a single item on the MIQY carries more weight, and may have led to more respondents being categorised as experiencing no mental image than would have on a measure with multiple items. Finally, the subgroup analyses were conducted on reduced sample sizes, and as such may be underpowered to detect smaller effect sizes. Further research with larger sample sizes should be conducted to overcome this.

### Research and clinical implications

Our findings demonstrated that baseline self‐perception was associated with future depressive symptoms, even over a relatively short period of 1 month. This aligns with theoretical models of depression, such as Beck's cognitive model and the self‐memory system, which identify self‐perception as an important contributor towards depressive symptoms (Beck, 1967; Conway, 2005). Interventions that aim to increase self‐perception could therefore be an effective way to reduce depressive symptomology in young people, aligning with the conclusions of previous research emphasising the need for interventions for depression to support young people to develop positive views of themselves (Dean et al., 2024). The associations between the experience of mental images of the self and depressive symptoms demonstrate that mental imagery may co‐occur alongside depression and potentially play a maintaining role. Clinically, practitioners may find it useful to assess young people's mental imagery of themselves and consider whether intervention may improve symptoms of depression.

Future research should investigate the use of a range of different measures and methodologies to assess mental imagery of the self in young people. Mental imagery is a multifaceted experience with an array of indices that vary across individuals. Whilst we focus on vividness and frequency in this study due to previous literature indicating their importance in the experience of youth depression, there are many other dimensions of the mental imagery experience that it may be beneficial to examine (e.g., controllability). A recent systematic review identified 21 self‐report measures assessing a range of mental‐imagery related processes, but found limited evidence of their reliability and validity (McIntyre et al., 2024). Additional research is needed to assess the psychometric properties of these measures to ensure that researchers have tools available to assess mental imagery. Furthermore, challenges exist in capturing and quantifying complex cognitive experiences like mental imagery. Qualitative research could be a useful avenue to fully explore the nuance and depth of young people's experiences of mental imagery, so that we can identify the aspects of mental imagery most relevant to depression in young people (e.g., Dean et al., 2025). Finally, at present mental imagery‐based interventions for depression do not always focus on the self and instead target mental imagery more broadly. It is important to investigate different types of mental imagery to determine their relative contributions towards the experience of depression. Understanding this could support the development of interventions that target types of mental imagery that contribute most towards depressive symptomology and boost the effectiveness of interventions. Experimental designs should therefore be used to investigate the utility of mental images of the self as a target for treating depression. Manipulation protocols should be used to test the effects of different techniques targeting mental images of the self on mood. These findings could support the identification of effective mental imagery‐based techniques to support young people with depression.

## CONCLUSIONS

This study provides evidence that self‐perception is associated with depressive symptoms cross‐sectionally and longitudinally in young people, contributing to our understanding about the role of self‐perception in the experience of depression. Whether an individual experiences a positive or negative mental image of themselves is also associated with depressive symptoms. Taken together, this research demonstrates that how young people view themselves, whether through their thoughts or their imagery, is a crucial part of depression. These results have important theoretical implications regarding the roles of self‐perception and mental images of the self in the development and maintenance of depression in young people.

## AUTHOR CONTRIBUTIONS

**Rebecca L. Dean**: Conceptualization; data curation; formal analysis; investigation; methodology; project administration; writing—original draft. **Victoria Pile**: Conceptualization; methodology; supervision; writing—review and editing. **Ellen J. Thompson**: Supervision; writing—review and editing. **Kathryn J. Lester**: Conceptualization; methodology; supervision; writing—review and editing. **Faith Orchard**: Conceptualization; methodology; supervision; writing—review and editing.

## CONFLICT OF INTEREST STATEMENT

The authors declare no conflicts of interest.

## ETHICAL CONSIDERATIONS

The study was granted ethical approval by the University of Sussex Science and Technology Cross‐Schools research ethics committee on August 1, 2022 (ER/RD416/4). All participants gave written informed consent to participate in this study. Participants aged under 16 required additional consent from a parent or carer.

## Supporting information

Supporting Information S1

## Data Availability

The data that support the findings of this study are available from the corresponding author upon reasonable request.

## References

[jcv270095-bib-0001] Bär, A. , Bär, H. E. , Schneider, M. , & Renner, F. (2023). The pupil as a window to the mind’s eye: Greater emotionality of episodic imagery than verbal visualisation of rewarding activities. Journal of Experimental Psychopathology, 14(2), 204380872311611. 10.1177/20438087231161176

[jcv270095-bib-0002] Bates, D. , Maechler, M. , Bolker, B. , & Walker, S. (2024). lme4: Linear mixed‐effects models using ‘Eigen’ and S4 (p. 1.1–35.4) [Dataset]. 10.32614/CRAN.package.lme4

[jcv270095-bib-0003] Beck, A. T. (1967). Depression. Harper and Row.

[jcv270095-bib-0004] Blackwell, S. E. (2023). Mental imagery and interpretational processing biases. In M. L. Woud (Ed.), Interpretational processing biases in emotional psychopathology: From experimental investigation to clinical practice (pp. 97–116). Springer International Publishing. 10.1007/978-3-031-23650-1_6

[jcv270095-bib-0005] Clayborne, Z. M. , Varin, M. , & Colman, I. (2019). Systematic review and meta‐analysis: Adolescent depression and long‐term psychosocial outcomes. Journal of the American Academy of Child & Adolescent Psychiatry, 58(1), 72–79. 10.1016/j.jaac.2018.07.896 30577941

[jcv270095-bib-0006] Conway, M. A. (2005). Memory and the self. Journal of Memory and Language, 53(4), 594–628. 10.1016/j.jml.2005.08.005

[jcv270095-bib-0007] Craske, M. G. , Dunn, B. D. , Meuret, A. E. , Rizvi, S. J. , & Taylor, C. T. (2024). Positive affect and reward processing in the treatment of depression, anxiety and trauma. Nature Reviews Psychology, 3(10), 665–685. 10.1038/s44159-024-00355-4

[jcv270095-bib-0008] Daly, M. (2022). Prevalence of depression among adolescents in the U.S. from 2009 to 2019: Analysis of trends by sex, race/ethnicity, and income. Journal of Adolescent Health, 70(3), 496–499. 10.1016/j.jadohealth.2021.08.026 34663534

[jcv270095-bib-0009] Dance, C. J. , Ipser, A. , & Simner, J. (2022). The prevalence of aphantasia (imagery weakness) in the general population. Consciousness and Cognition, 97, 103243. 10.1016/j.concog.2021.103243 34872033

[jcv270095-bib-0010] D’Argembeau, A. (2021). Memory, future thinking, and the self. In honour of Martial Van Der Linden. Psychologica Belgica, 61(1), 274–283. 10.5334/pb.1074 34611494 PMC8447971

[jcv270095-bib-0011] Dean, R. L. , Lester, K. J. , Grant, E. , Field, A. P. , Orchard, F. , & Pile, V. (2024). The impact of interventions for depression on self‐perceptions in young people: A systematic review & meta‐analysis. Clinical Psychology Review, 115, 102521. 10.1016/j.cpr.2024.102521 39622116

[jcv270095-bib-0012] Dean, R. L. , Orchard, F. , Pile, V. , & Lester, K. J. (2025). “When I picture myself, I just see black and white and dull”: A photo‐elicitation study exploring mental images of the self in young people with depression. BMC Psychiatry, 25(1), 642. 10.1186/s12888-025-07072-z 40597068 PMC12220442

[jcv270095-bib-0013] Ebesutani, C. , Korathu‐Larson, P. , Nakamura, B. J. , Higa‐McMillan, C. , & Chorpita, B. (2017). The Revised Child Anxiety and Depression Scale 25–Parent Version: Scale development and validation in a school‐based and clinical sample. Assessment, 24(6), 712–728. 10.1177/1073191115627012 26834091

[jcv270095-bib-0014] Ebesutani, C. , Reise, S. P. , Chorpita, B. F. , Ale, C. , Regan, J. , Young, J. , Higa‐McMillan, C. , & Weisz, J. R. (2012). The Revised Child Anxiety and Depression Scale‐Short Version: Scale reduction via exploratory bifactor modeling of the broad anxiety factor. Psychological Assessment, 24(4), 833–845. 10.1037/a0027283 22329531

[jcv270095-bib-0015] Eckshtain, D. , Kuppens, S. , Ugueto, A. , Ng, M. Y. , Vaughn‐Coaxum, R. , Corteselli, K. , & Weisz, J. R. (2020). Meta‐analysis: 13‐year follow‐up of psychotherapy effects on youth depression. Journal of the American Academy of Child & Adolescent Psychiatry, 59(1), 45–63. 10.1016/j.jaac.2019.04.002 31004739

[jcv270095-bib-0016] Fennell, M. J. V. (1998). Cognitive therapy in the treatment of low self‐esteem. Advances in Psychiatric Treatment, 4(5), 296–304. 10.1192/apt.4.5.296

[jcv270095-bib-0017] Gulyás, E. , Gombos, F. , Sütöri, S. , Lovas, A. , Ziman, G. , & Kovács, I. (2022). Visual imagery vividness declines across the lifespan. Cortex, 154, 365–374. 10.1016/j.cortex.2022.06.011 35921690

[jcv270095-bib-0018] Hackmann, A. , Clark, D. M. , & McManus, F. (2000). Recurrent images and early memories in social phobia. Behaviour Research and Therapy, 38(6), 601–610. 10.1016/S0005-7967(99)00161-8 10846808

[jcv270095-bib-0019] Hards, E. , Orchard, F. , & Reynolds, S. (2023). ‘I am tired, sad and kind’: Self‐evaluation and symptoms of depression in adolescents. Child and Adolescent Psychiatry and Mental Health, 17(1), 126. 10.1186/s13034-023-00661-4 37941014 PMC10633984

[jcv270095-bib-0020] Harter, S. (2012). Self‐perception profile for adolescents: Manual and questionnaires. University of Denver.

[jcv270095-bib-0021] Holmes, E. A. , Blackwell, S. E. , Burnett Heyes, S. , Renner, F. , & Raes, F. (2016). Mental imagery in depression: Phenomenology, potential mechanisms, and treatment implications. Annual Review of Clinical Psychology, 12(1), 249–280. 10.1146/annurev-clinpsy-021815-092925 26772205

[jcv270095-bib-0022] Johnson, D. , Dupuis, G. , Piche, J. , Clayborne, Z. , & Colman, I. (2018). Adult mental health outcomes of adolescent depression: A systematic review. Depression and Anxiety, 35(8), 700–716. 10.1002/da.22777 29878410

[jcv270095-bib-0023] Kertz, S. J. , Petersen, D. R. , & Stevens, K. T. (2019). Cognitive and attentional vulnerability to depression in youth: A review. Clinical Psychology Review, 71, 63–77. 10.1016/j.cpr.2019.01.004 30732975

[jcv270095-bib-0024] Kessler, R. C. , Berglund, P. , Demler, O. , Jin, R. , Merikangas, K. R. , & Walters, E. E. (2005). Lifetime prevalence and age‐of‐onset distributions of DSM‐IV disorders in the National Comorbidity Survey Replication. Archives of General Psychiatry, 62(6), 593–602. 10.1001/archpsyc.62.6.593 15939837

[jcv270095-bib-0025] Kosslyn, S. M. , Chabris, C. , Shephard, J. , & Thompson, W. (1998). Spontaneous use of imagery Scale. APA PsycTests. 10.1037/t57899-000

[jcv270095-bib-0026] Kosslyn, S. M. , Ganis, G. , & Thompson, W. L. (2001). Neural foundations of imagery. Nature Reviews Neuroscience, 2(9), 9. 10.1038/35090055 11533731

[jcv270095-bib-0027] Kuyken, W. , & Howell, R. (2006). Facets of autobiographical memory in adolescents with major depressive disorder and never‐depressed controls. Cognition & Emotion, 20(3–4), 466–487. 10.1080/02699930500342639 26529216

[jcv270095-bib-0028] Kuznetsova, A. , Bruun Brockhoff, P. , & Haubo Bojesen Christensen, R. (2020). lmerTest: Tests in linear mixed effects models (p. 3.1–3) [Dataset]. 10.32614/CRAN.package.lmerTest

[jcv270095-bib-0029] MacLeod, A. K. , & Byrne, A. (1996). Anxiety, depression, and the anticipation of future positive and negative experiences. Journal of Abnormal Psychology, 105(2), 286–289. 10.1037/0021-843X.105.2.286 8723011

[jcv270095-bib-0030] Marciniak, M. A. , Shanahan, L. , Binder, H. , Kalisch, R. , & Kleim, B. (2023). Positive prospective mental imagery characteristics in young adults and their associations with depressive symptoms. Cognitive Therapy and Research, 47(4), 695–706. 10.1007/s10608-023-10378-5 PMC10140715 37363749

[jcv270095-bib-0031] Marks, D. F. (1973). Visual imagery differences in the recall of pictures. British Journal of Psychology, 64(1), 17–24. 10.1111/j.2044-8295.1973.tb01322.x 4742442

[jcv270095-bib-0032] McGrath, J. J. , Al‐Hamzawi, A. , Alonso, J. , Altwaijri, Y. , Andrade, L. H. , Bromet, E. J. , Bruffaerts, R. , Almeida, J. M. C. de , Chardoul, S. , Chiu, W. T. , Degenhardt, L. , Demler, O. V. , Ferry, F. , Gureje, O. , Haro, J. M. , Karam, E. G. , Karam, G. , Khaled, S. M. , Kovess‐Masfety, V. , & Zaslavsky, A. M. (2023). Age of onset and cumulative risk of mental disorders: A cross‐national analysis of population surveys from 29 countries. The Lancet Psychiatry, 668–681. 10.1016/S2215-0366(23)00193-1 37531964 PMC10529120

[jcv270095-bib-0033] McIntyre, S. A. , Richardson, J. , Carroll, S. , O’Kirwan, S. , Williams, C. , & Pile, V. (2024). Measures of mental imagery in emotional disorders: A COSMIN systematic review of psychometric properties. Clinical Psychology Review, 113, 102470. 10.1016/j.cpr.2024.102470 39180928

[jcv270095-bib-0034] McKenzie, K. , Murray, A. , Freeston, M. , Whelan, K. , & Rodgers, J. (2019). Validation of the Revised Children’s Anxiety and Depression Scales (RCADS) and RCADS short forms adapted for adults. Journal of Affective Disorders, 245, 200–204. 10.1016/j.jad.2018.10.362 30399524

[jcv270095-bib-0035] Meiser‐Stedman, R. , Dalgleish, T. , Yule, W. , & Smith, P. (2012). Intrusive memories and depression following recent non‐traumatic negative life events in adolescents. Journal of Affective Disorders, 137(1), 70–78. 10.1016/j.jad.2011.12.020 22244376

[jcv270095-bib-0036] Neeman, J. , & Harter, S. (2012). Self‐perception profile for college students: Manual and questionnaires. University of Denver.

[jcv270095-bib-0037] Ng, A. S. , Abbott, M. J. , & Hunt, C. (2014). The effect of self‐imagery on symptoms and processes in social anxiety: A systematic review. Clinical Psychology Review, 34(8), 620–633. 10.1016/j.cpr.2014.09.003 25455626

[jcv270095-bib-0038] Ng, M. Y. , & Weisz, J. R. (2016). Annual research review: Building a science of personalized intervention for youth mental health. Journal of Child Psychology and Psychiatry, 57(3), 216–236. 10.1111/jcpp.12470 26467325 PMC4760855

[jcv270095-bib-0039] Orchard, F. , Pass, L. , & Reynolds, S. (2019). ‘I am worthless and kind’; the specificity of positive and negative self‐evaluation in adolescent depression. British Journal of Clinical Psychology, 58(3), 260–273. 10.1111/bjc.12215 30556150 PMC6767166

[jcv270095-bib-0040] Orchard, F. , Westbrook, J. , Gee, B. , Clarke, T. , Allan, S. , & Pass, L. (2021). Self‐evaluation as an active ingredient in the experience and treatment of adolescent depression; an integrated scoping review with expert advisory input. BMC Psychiatry, 21(1), 603. 10.1186/s12888-021-03585-5 34861833 PMC8641228

[jcv270095-bib-0041] Pearson, J. , Naselaris, T. , Holmes, E. A. , & Kosslyn, S. M. (2015). Mental imagery: Functional mechanisms and clinical applications. Trends in Cognitive Sciences, 19(10), 590–602. 10.1016/j.tics.2015.08.003 26412097 PMC4595480

[jcv270095-bib-0042] Petito, A. , Pop, T. L. , Namazova‐Baranova, L. , Mestrovic, J. , Nigri, L. , Vural, M. , Sacco, M. , Giardino, I. , Ferrara, P. , & Pettoello‐Mantovani, M. (2020). The burden of depression in adolescents and the importance of early recognition. The Journal of Pediatrics, 218, 265–267.e1. 10.1016/j.jpeds.2019.12.003 31932020

[jcv270095-bib-0043] Pile, V. , & Lau, J. Y. F. (2018). Looking forward to the future: Impoverished vividness for positive prospective events characterises low mood in adolescence. Journal of Affective Disorders, 238, 269–276. 10.1016/j.jad.2018.05.032 29894932

[jcv270095-bib-0044] Pile, V. , & Lau, J. Y. F. (2020). Intrusive images of a distressing future: Links between prospective mental imagery, generalized anxiety and a tendency to suppress emotional experience in youth. Behaviour Research and Therapy, 124, 103508. 10.1016/j.brat.2019.103508 31855697

[jcv270095-bib-0045] Pile, V. , Williamson, G. , Saunders, A. , Holmes, E. A. , & Lau, J. Y. F. (2021). Harnessing emotional mental imagery to reduce anxiety and depression in young people: An integrative review of progress and promise. The Lancet Psychiatry, 8(9), 836–852. 10.1016/S2215-0366(21)00195-4 34419188

[jcv270095-bib-0046] Schreiber, F. , & Steil, R. (2013). Haunting self‐images? The role of negative self‐images in adolescent social anxiety disorder. Journal of Behavior Therapy and Experimental Psychiatry, 44(2), 158–164. 10.1016/j.jbtep.2012.10.003 23207962

[jcv270095-bib-0047] Schwarz, S. , Feike, M. , & Stangier, U. (2021). Mental imagery and social pain in adolescents—Analysis of imagery characteristics and perspective—A pilot study. Children, 8(12), 1160. 10.3390/children8121160 34943356 PMC8700563

[jcv270095-bib-0048] Schwarz, S. , Grasmann, D. , Schreiber, F. , & Stangier, U. (2020). Mental imagery and its relevance for psychopathology and psychological treatment in children and adolescents: A systematic review. International Journal of Cognitive Therapy, 13(4), 303–327. 10.1007/s41811-020-00092-5

[jcv270095-bib-0049] Schwarz, S. , & Schreiber, F. (2016). Fragebogen zur Erfassung mentaler Bilder bei Jugendlichen (FEMB‐J) [Unpublished manuscript].

[jcv270095-bib-0050] Schwarz, S. , & Stangier, U. (2023). Contents and characteristics of mental imagery and their association with emotional intensity in adolescents: A pilot study. Journal of Rational‐Emotive and Cognitive‐Behavior Therapy, 41(4), 838–855. 10.1007/s10942-023-00515-0

[jcv270095-bib-0051] Shorey, S. , Ng, E. D. , & Wong, C. H. J. (2022). Global prevalence of depression and elevated depressive symptoms among adolescents: A systematic review and meta‐analysis. British Journal of Clinical Psychology, 61(2), 287–305. 10.1111/bjc.12333 34569066

[jcv270095-bib-0052] Stopa, L. , & Beck, J. S. (2021). Imagery in cognitive‐behavioral therapy. Guilford Publications. http://ebookcentral.proquest.com/lib/suss/detail.action?docID=6637369

[jcv270095-bib-0053] Voogd, E. L. de , Hullu, E. de , Heyes, S. B. , Blackwell, S. E. , Wiers, R. W. , & Salemink, E. (2017). Imagine the bright side of life: A randomized controlled trial of two types of interpretation bias modification procedure targeting adolescent anxiety and depression. PLoS One, 12(7), e0181147. 10.1371/journal.pone.0181147 28715495 PMC5513454

[jcv270095-bib-0054] Weisenburger, R. L. , Dainer‐Best, J. , Zisser, M. , McNamara, M. E. , & Beevers, C. G. (2024). Negative self‐referent cognition predicts future depression symptom change: An intensive sampling approach. Cognition & Emotion, 1–15. 10.1080/02699931.2024.2400298 39264587

[jcv270095-bib-0055] Werner‐Seidler, A. , & Moulds, M. L. (2011). Autobiographical memory characteristics in depression vulnerability: Formerly depressed individuals recall less vivid positive memories. Cognition & Emotion, 25(6), 1087–1103. 10.1080/02699931.2010.531007 21895571

[jcv270095-bib-0056] Werner‐Seidler, A. , & Moulds, M. L. (2012). Characteristics of self‐defining memory in depression vulnerability. Memory, 20(8), 935–948. 10.1080/09658211.2012.712702 22900963

[jcv270095-bib-0057] Weßlau, C. , & Steil, R. (2014). Visual mental imagery in psychopathology—Implications for the maintenance and treatment of depression. Clinical Psychology Review, 34(4), 273–281. 10.1016/j.cpr.2014.03.001 24727643

[jcv270095-bib-0058] Wichstraum, L. (1995). Harter’s Self‐Perception Profile for Adolescents: Reliability, validity, and evaluation of the question format. Journal of Personality Assessment, 65(1), 100–116. 10.1207/s15327752jpa6501_8 7643294

[jcv270095-bib-0059] Wittchen, H.‐U. , Kessler, R. C. , Pfister, H. , Höfler, M. , & Lieb, R. (2000). Why do people with anxiety disorders become depressed? A prospective‐longitudinal community study. Acta Psychiatrica Scandinavica, 102(s406), 14–23. 10.1111/j.0065-1591.2000.acp29-03.x 11131466

[jcv270095-bib-0060] World Health Organization . (2024). Adolescent and young adult health. https://www.who.int/news‐room/fact‐sheets/detail/adolescents‐health‐risks‐and‐solutions

