# Supplementary material for: A longitudinal exploration of self‐perception, mental images of the self, and depression in young people
Source: JCPP Adv. 2026 Feb 10;6(3):e70095. doi: 10.1002/jcv2.70095 (PMC13339219; doi:10.1002/jcv2.70095)
Supplement: Supplementary file 1 [file JCV2-6-e70095-s001.docx]

**A longitudinal exploration of self-perception, mental images of the self, and depression in young people**

**Supporting Information**

*Table S1. SUIS descriptive statistics*

|  | | **Baseline** | | | **Follow-up** | | |
| --- | --- | --- | --- | --- | --- | --- | --- |
| **Clinical characteristics** | | **School**  ***(n=313)*** | **University *(n=483)*** | **Full sample *(n=796)*** | **School**  ***(n=215)*** | **University sample *(n=346)*** | **Full sample *(n=561)*** |
| **SUIS score** | | | | |  |  |  |
|  | Mean | 22.1 (5.47) | 23.4 (4.53) | 22.9 (4.95) | 26.2 (4.04) | 23.4 (4.56) | 24.5 (4.57) |
|  | Median [min, max] | 23.0 [7.0, 35.0] | 24.0 [9.0, 34.0] | 23.0 [7.0, 35.0) | 26.0 [14.0, 35.0] | 24.0 [9.0, 35.0] | 25.0 [9.0, 35.0] |
|  | Missing | 10 (3.2%) | 10 (2.1%) | 20 (2.5%) | 5 (2.3%) | 3 (0.9%) | 8 (1.4%) |

Table S2. Distribution of participant ages by institution

| **School/**  **Age** | **1**  **(N=38)** | **2**  **(N=61)** | **3**  **(N=29)** | **4**  **(N=11)** | **5**  **(N=33)** | **6**  **(N=44)** | **7**  **(N=63)** | **8**  **(N=34)** | **University**  **(N=483)** | **Total**  **(N=796)** |
| --- | --- | --- | --- | --- | --- | --- | --- | --- | --- | --- |
| 12 | 0 (0%) | 0 (0%) | 0 (0%) | 5 (45.5%) | 0 (0%) | 0 (0%) | 0 (0%) | 5 (14.7%) | 0 (0%) | 10 (1.3%) |
| 13 | 0 (0%) | 0 (0%) | 0 (0%) | 6 (54.5%) | 0 (0%) | 0 (0%) | 0 (0%) | 9 (26.5%) | 0 (0%) | 15 (1.9%) |
| 14 | 0 (0%) | 26 (42.6%) | 9 (31.0%) | 0 (0%) | 0 (0%) | 1 (2.3%) | 0 (0%) | 2 (5.9%) | 0 (0%) | 38 (4.8%) |
| 15 | 0 (0%) | 30(49.2%) | 1 (3.4%) | 0 (0%) | 0 (0%) | 31 (70.5%) | 0 (0%) | 2 (5.9%) | 0 (0%) | 64 (8.0%) |
| 16 | 0 (0%) | 5 (8.2%) | 3 (10.3%) | 0 (0%) | 7 (21.2%) | 12 (27.3%) | 25 (39.7%) | 6 (17.6%) | 0 (0%) | 58 (7.3%) |
| 17 | 26 (68.4%) | 0 (0%) | 11 (37.9%) | 0 (0%) | 24 (72.7%) | 0 (0%) | 30 (47.6%) | 8 (23.5%) | 2 (0.4%) | 101 (12.7%) |
| 18 | 12 (31.6%) | 0 (0%) | 5 (17.2%) | 0 (0%) | 2 (6.1%) | 0 (0%) | 7 (11.1%) | 2 (5.9%) | 155 (32.1%) | 183 (23.0%) |
| 19 | 0 (0%) | 0 (0%) | 0 (0%) | 0 (0%) | 0 (0%) | 0 (0%) | 1 (1.6%) | 0 (0%) | 172 (35.6%) | 173 (21.7%) |
| 20 | 0 (0%) | 0 (0%) | 0 (0%) | 0 (0%) | 0 (0%) | 0 (0%) | 0 (0%) | 0 (0%) | 97 (20.1%) | 97 (12.2%) |
| 21 | 0 (0%) | 0 (0%) | 0 (0%) | 0 (0%) | 0 (0%) | 0 (0%) | 0 (0%) | 0 (0%) | 32 (6.6%) | 32 (4.0%) |
| 22 | 0 (0%) | 0 (0%) | 0 (0%) | 0 (0%) | 0 (0%) | 0 (0%) | 0 (0%) | 0 (0%) | 15 (3.1%) | 15 (1.9%) |
| 23 | 0 (0%) | 0 (0%) | 0 (0%) | 0 (0%) | 0 (0%) | 0 (0%) | 0 (0%) | 0 (0%) | 6 (1.2%) | 6 (0.8%) |
| 24 | 0 (0%) | 0 (0%) | 0 (0%) | 0 (0%) | 0 (0%) | 0 (0%) | 0 (0%) | 0 (0%) | 4 (0.8%) | 4 (0.5%) |

*Table S3. Self-perception descriptive statistics*

|  | | **Baseline** | | **Follow-up** | |
| --- | --- | --- | --- | --- | --- |
|  | | **School**  ***(n=313)*** | **University**  ***(n=483)*** | **School**  ***(n=215)*** | **University**  ***(n=346)*** |
| **Self-Perception** | | | | | |
|  | Mean (SD) | 12.9 (3.18) | 14.8 (3.73) | 13.4 (3.22) | 15.0 (3.66) |
|  | Median [min, max] | 13.0 [6.0, 20.0] | 15.0 [6.00, 24.0] | 14.0 [5.0, 24.0] | 15.0 [6.00, 24.0] |
|  | Missing | 8 (2.6%) | 13 (2.7%) | 4 (1.9%) | 4 (1.2%) |

*Note: that the school and university samples completed different age-appropriate versions of the Harter Self-Perception Scale self-worth subscale with a different number of items (5 for the school sample, 6 for the university sample), therefore the means for the University sample are higher. We have not examined descriptive statistics for the full sample for this reason, and this is accounted for in analyses by using Z-scores, as detailed in the Methods.*

*Table S4. Experience of key mental imagery characteristics by institution type*

|  | | **Baseline** | | | **Follow-up** | | | | | |  |
| --- | --- | --- | --- | --- | --- | --- | --- | --- | --- | --- | --- |
| **Clinical characteristics** | | **School**  ***(n=313)*** | **University *(n=483)*** | **Full sample *(n=796)*** | **School**  ***(n=215)*** | | **University sample *(n=346)*** | | **Full sample *(n=561)*** | |  |
| **Whether participants experienced positive mental imagery** | | | | |  | |  | |  | |  |
|  | Yes | 195 (62.3%) | 340 (70.4%) | 535 (67.2%) | 141 (65.6%) | | 243 (70.2%) | | 384 (68.4%) | |  |
|  | No | 108 (34.5%) | 138 (28.6%) | 246 (30.9%) | 69 (32.1%) | | 101 (29.2%) | | 170 (30.3%) | |  |
|  | Missing | 10 (3.2%) | 5 (1.0%) | 15 (1.9%) | 5 (2.3%) | | 2 (0.6%) | | 7 (1.2%) | |  |
| **Positive mental imagery frequency score** | | | | |  | |  | |  | |  |
|  | Always | 10 (3.2%) | 11 (2.3%) | 21 (2.6%) | 5 (2.3%) | | 4 (1.2%) | | 9 (1.6%) | |  |
|  | Often | 43 (13.7%) | 61 (12.6%) | 104 (13.1%) | 26 (12.1%) | | 54 (15.6%) | | 80 (14.3%) | |  |
|  | Sometimes | 58 (18.5%) | 120 (24.8%) | 178 (22.4%) | 58 (27.0%) | | 79 (22.8%) | | 137 (24.4%) | |  |
|  | Rarely | 40 (12.8%) | 81 (16.8%) | 121 (15.2%) | 28 (13.0%) | | 68 (19.7%) | | 96 (17.1%) | |  |
|  | Almost never (only when I am asked) | 36 (11.5%) | 61 (12.6%) | 97 (12.2%) | 20 (9.3%) | | 36 (10.4%) | | 56 (10.0%) | |  |
|  | Never | 108 (34.5%) | 138 (28.6%) | 246 (30.9%) | 69 (32.1%) | | 101 (29.2%) | | 170 (30.3%) | |  |
|  | Missing | 18 (5.8%) | 11 (2.3%) | 29 (3.6%) | 9 (4.2%) | | 4 (1.2%) | | 13 (2.3%) | |  |
| **Positive mental imagery frequency (categorised)** | | | | |  | |  | |  | |  |
|  | High | 111 (35.5%) | 192 (39.8%) | 303 (38.1%) | 89 (41.4%) | | 137 (39.6%) | | 226 (40.3%) | |  |
|  | Low | 184 (58.8%) | 280 (58.0%) | 464 (58.3%) | 117 (54.4%) | | 205 (59.2%) | | 322 (57.4%) | |  |
|  | Missing | 18 (5.8%) | 11 (2.3%) | 29 (3.6%) | 9 (4.2%) | | 4 (1.2%) | | 13 (2.3%) | |  |
| **Positive mental imagery vividness score** | | | | |  | |  | |  | |  |
|  | Perfectly clear and as vivid as in real-life | 26 (8.3%) | 38 (7.9%) | 64 (8.0%) | 18 (8.4%) | | 26 (7.5%) | | 44 (7.8%) | |  |
|  | Clear and nearly as vivid as in real-life | 58 (18.5%) | 114 (23.6%) | 172 (21.6%) | 44 (20.5%) | | 80 (23.1%) | | 124 (22.1%) | |  |
|  | Moderately clear and vivid | 78 (24.9%) | 137 (28.4%) | 215 (27.0%) | 51 (23.7%) | | 104 (30.1%) | | 155 (27.6%) | |  |
|  | Fuzzy and hazy | 19 (6.1%) | 40 (8.3%) | 59 (7.4%) | 16 (7.4%) | | 28 (8.1%) | | 44 (7.8%) | |  |
|  | No mental image at all | 116 (37.1%) | 145 (30.0%) | 261 (32.8%) | 78 (36.3%) | | 104 (30.1%) | | 182 (32.4%) | |  |
|  | Missing | 16 (5.1%) | 9 (1.9%) | 25 (3.1%) | 8 (3.7%) | | 4 (1.2%) | | 12 (2.1%) | |  |
| **Positive mental imagery vividness (categorised)** | | | | |  | |  | |  | |  |
|  | High | 162 (51.8%) | 289 (59.8%) | 451 (56.7%) | 113 (52.6%) | | 210 (60.7%) | | 323 (57.6%) | |  |
|  | Low | 135 (43.1%) | 185 (38.3%) | 320 (40.2%) | 94 (43.7%) | | 132 (38.2%) | | 226 (40.3%) | |  |
|  | Missing | 16 (5.1%) | 9 (1.9%) | 25 (3.1%) | 8 (3.7%) | | 4 (1.2%) | | 12 (2.1%) | |  |
| **Whether participants experienced negative mental imagery** | | | | | |  | |  | |  | |
|  | Yes | 170 (54.3%) | 322 (66.7%) | 492 (61.8%) | 98 (45.6%) | | 212 (61.3%) | | 310 (55.3%) | |  |
|  | No | 134 (42.8%) | 154 (31.9%) | 288 (36.2%) | 116 (54.0%) | | 132 (38.2%) | | 248 (44.2%) | |  |
|  | Missing | 9 (2.9%) | 7 (1.4%) | 16 (2.0%) | 1 (0.5%) | | 2 (0.6%) | | 3 (0.5%) | |  |
| **Negative mental imagery frequency score** | | | | |  | |  | |  | |  |
|  | Always | 8 (2.6%) | 12 (2.5%) | 20 (2.5%) | 8 (3.7%) | | 8 (2.3%) | | 16 (2.9%) | |  |
|  | Often | 55 (17.6%) | 77 (15.9%) | 132 (16.6%) | 32 (14.9%) | | 46 (13.3%) | | 78 (13.9%) | |  |
|  | Sometimes | 45 (14.4%) | 130 (26.9%) | 175 (22.0%) | 26 (12.1%) | | 81 (23.4%) | | 107 (19.1%) | |  |
|  | Rarely | 24 (7.7%) | 63 (13.0%) | 87 (10.9%) | 21 (9.8%) | | 49 (14.2%) | | 70 (12.5%) | |  |
|  | Almost never (only if I am asked) | 27 (8.6%) | 35 (7.2%) | 62 (7.8%) | 6 (2.8%) | | 26 (7.5%) | | 32 (5.7%) | |  |
|  | Never | 134 (42.8%) | 154 (31.9%) | 288 (36.2%) | 116 (54.0%) | | 132 (38.2%) | | 248 (44.2%) | |  |
|  | Missing | 20 (6.4%) | 12 (2.5%) | 32 (4.0%) | 6 (2.8%) | | 4 (1.2%) | | 10 (1.8%) | |  |
| **Negative mental imagery frequency (categorised)** | | | | |  | |  | |  | |  |
|  | High | 108 (34.5%) | 219 (45.3%) | 327 (41.1%) | 66 (30.7%) | | 135 (39.0%) | | 201 (35.8%) | |  |
|  | Low | 185 (59.1%) | 252 (52.2%) | 437 (54.9%) | 143 (66.5%) | | 207 (59.8%) | | 350 (62.4%) | |  |
|  | Missing | 20 (6.4%) | 12 (2.5%) | 32 (4.0%) | 6 (2.8%) | | 4 (1.2%) | | 10 (1.8%) | |  |
| **Negative mental imagery vividness score** | | | | | | | | | | |  |
|  | Perfectly clear and as vivid as in real-life | 21 (6.7%) | 25 (5.2%) | 46 (5.8%) | 11 (5.1%) | | 17 (4.9%) | | 28 (5.0%) | |  |
|  | Clear and nearly as vivid as in real-life | 44 (14.1%) | 94 (19.5%) | 138 (17.3%) | 21 (9.8%) | | 53 (15.3%) | | 74 (13.2%) | |  |
|  | Moderately clear and vivid | 57 (18.2%) | 133 (27.5%) | 190 (23.9%) | 42 (19.5%) | | 93 (26.9%) | | 135 (24.1%) | |  |
|  | Fuzzy and hazy | 27 (8.6%) | 60 (12.4%) | 87 (10.9%) | 16 (7.4%) | | 38 (11.0%) | | 54 (9.6%) | |  |
|  | No mental image at all | 145 (46.3%) | 159 (32.9%) | 304 (38.2%) | 120 (55.8%) | | 141 (40.8%) | | 261 (46.5%) | |  |
|  | Missing | 19 (6.1%) | 12 (2.5%) | 31 (3.9%) | 5 (2.3%) | | 4 (1.2%) | | 9 (1.6%) | |  |
| **Negative mental imagery vividness (categorised)** | | | | |  | |  | |  | |  |
|  | High | 122 (39.0%) | 252 (52.2%) | 374 (47.0%) | 74 (34.4%) | | 163 (47.1%) | | 237 (42.2%) | |  |
|  | Low | 172 (55.0%) | 219 (45.3%) | 391 (49.1%) | 136 (63.3%) | | 179 (51.7%) | | 315 (56.1%) | |  |
|  | Missing | 19 (6.1%) | 12 (2.5%) | 31 (3.9%) | 5 (2.3%) | | 4 (1.2%) | | 9 (1.6%) | |  |

*Note: A score of three and above out of five corresponded to a ‘high’ level of vividness or frequency, and a score of two or lower was considered ‘low’.*

*Table S5. Summary of models predicting depression and self-perception (school sample only)*

|  |  | *Summary of models predicting depression and self-perception*  Independent variables | | | | |
| --- | --- | --- | --- | --- | --- | --- |
|  |  | Self-perception | Negative mental image frequency | Negative mental image vividness | Positive mental image frequency | Positive mental image vividness |
| Dependent variables | | $b$ (95% CI) | $b$ (95% CI) | $b$ (95% CI) | $b$ (95% CI) | $b$ (95% CI) |
| ***Baseline*** | Depression | **-2.20 (-2.70, -1.70)*** | **0.36 (0.05, 0.68)** | 0.23 (-0.39, 0.87) | **-0.49 (-0.76, -0.22)*** | -0.15 (-0.66, 0.36) |
|  | Self-perception |  | **-0.10 (-0.16, -0.04)** | 0.06 (-0.06, 0.18)* | **0.11 (0.06, 0.16)** | **0.14 (0.03, 0.24)** |
| ***Follow-up*** | Depression | -0.63 (-1.33, 0.07) | -0.01 (-0.33, 0.31) | -0.30 (-0.93, 0.33) | 0.06 (-0.23, 0.36) | 0.22 (-0.36, 0.79) |
|  | Self-perception |  | 0.00 (-0.05, 0.05) | -0.07 (-0.18, 0.02)* | -0.01 (-0.06, 0.04) | -0.04 (-0.13, 0.05)* |
| ***Moderation***  ***Follow-up*** | Depression (Self-perception x mental image characteristic) |  | 0.38 (-0.77, 1.55) | 0.50 (-1.13, 2.13) | -0.06 (-1.09, 0.96) | -0.83 (-2.40, 0.75) |

*Note: Models with mental imagery vividness as an independent variable use data only from participants who reported experiencing a mental image of themselves and models with mental image frequency use imputed data. Baseline models were adjusted using gender, ethnicity, age, and anxiety. Follow-up models were further adjusted using time and baseline outcome. Models include a random effect of school unless indicated with *, and these models are also adjusted by age. Coefficients in bold are where the 95% CIs do not cross the null. Beta coefficients are unstandardised, except where self-perception was included in the models due to use of Z-scores.*

*Table S6. Summary of models predicting depression and self-perception (university sample only)*

|  |  | *Summary of models predicting depression and self-perception*  Independent variables | | | | |
| --- | --- | --- | --- | --- | --- | --- |
|  |  | Self-perception | Negative mental image frequency | Negative mental image vividness | Positive mental image frequency | Positive mental image vividness |
| Dependent variables | | $b$ (95% CI) | $b$ (95% CI) | $b$ (95% CI) | $b$ (95% CI) | $b$ (95% CI) |
| ***Baseline*** | Depression | **-2.76 (-3.13, -2.39)** | **0.36 (0.11, 0.61)** | -0.19 (-0.72, 0.33) | **-0.65 (-0.89, -0.40)** | -0.16 (-0.64, 0.31) |
|  | Self-perception |  | **-0.10 (-0.15, -0.05)** | -0.01 (-0.09, 0.11) | **0.13 (0.08, 0.18)** | 0.04 (-0.05, 0.13) |
| ***Follow-up*** | Depression | **-0.96 (-1.44, -0.49)** | 0.18 (-0.05, 0.42) | 0.07 (-0.41, 0.54) | -0.07 (-0.30, 0.17) | -0.28 (-0.70, 0.14) |
|  | Self-perception |  | -0.02(-0.07, 0.02) | 0.01(-0.07, 0.09) | **0.05 (0.01, 0.10)** | **0.09 (0.01, 0.16)** |
| ***Moderation***  ***Follow-up*** | Depression (Self-perception x mental image characteristic) |  | -0.04 (-0.76, 0.67) | 0.63 (-0.55, 1.81) | -0.21 (-0.91, 0.48) | 0.46 (-0.71, 1.63) |

*Note: Baseline models were adjusted using gender, ethnicity, age, and anxiety. Follow-up models were further adjusted using time and baseline outcome. Coefficients in bold are where the 95% CIs do not cross the null. Beta coefficients are unstandardised, except where self-perception was included in the models due to use of Z-scores.*

**Appendix S1. Mental Imagery Questionnaire for Youths (MIQ-Y) translation and adaptation process**

The German version of the MIQ-Y was provided by the authors of the questionnaire (Schwarz and colleagues). The questionnaire was translated into English by a PhD colleague of the lead author at the University of Sussex School of Psychology who was fluent in both English and German. The questionnaire was translated into English, and then checked by both the translator and lead author to check that the wording and grammar would make sense to native English speakers. References to mental images ‘of yourself’ were then added due to the focus of the study on mental images of the self.
